# Transfusion in Anæmia

**Published:** 1866-10

**Authors:** W. W. Myers

**Affiliations:** Pittsburg, Pennsylvania


*Transfusion in Anæmia.* By W. W. MYERS, M. D., Pittsburg, Pennsylvania.

February 5th, 1866.—Was called to see Henry, son of Wm. M., æt. five years, of Wineberry alley. Found him suffering with vertigo, faintishness, palpitation, and an impaired action of the organs generally; especially the stomach and bowels; digestion being deranged, with flatulency, constipation, etc. The face, the hands, and the general surface were pallid, and slightly waxen or icterode in their hue, clearly indicating the presence of general anæmia. Upon ausculating over the pulmonary and subclavian arteries, a bellowing sound was distinctly perceived, which is to be attributed to a certain diminution in the proportion of the globules. This is due to an impoverishment of these globules, inasmuch as it is not heard when the fibrin alone is diminished in quantity. The patient had been treated for some weeks previously for hepatitis, but the existence of such I was unable to detect. He was placed upon ferri pulvis, of which grains three were taken thrice daily, *after meals*, because, as every one knows, the gastric juice of the empty stomach is *alkaline;* during digestion it is *acid;* this acts favorably toward dissolving the iron, while the other renders it inert. A small allowance of wine was permitted, and a generous diet advised.

No apparent improvement having taken place by March 27th, the following was determined upon. Having taken some six or eight ounces of blood a few days previously, from a healthy male laboring under plethora, and this blood having been preserved from contact with atmospheric air, it was decided to transfuse a part of it into the patient. Every thing being in readiness, the median basilic was freely displayed, and an incision made down to it an inch in length; a probe was then passed beneath it, at the lower part of the incision; a small opening was then made in the vein, immediately beyond the probe, of sufficient size to admit the beak of the syringe—which having been previously warmed—was filled with this blood, it also having been heated to a temperature of about 95°. It was inserted, and ℥ iij. injected; it was refilled, and repeated. This quantity was deemed sufficient, for in the extreme feebleness to which the vital action of all the tissues is reduced in cases of protracted anæmia, it is desirable that the supply of blood should be very gradual, lest the action of any vital organ should be impaired by a sudden congestion of its tissues. The greatest possible caution was exercised to prevent the entrance of atmospheric air into the vein, the smallest quantity of which would prove destructive. The expression of the countenance and state of the pulse were carefully watched, but no untoward symptoms arose. A compress and bandage were placed over the orifice, and an opiate administered. The following was ordered:

℞ Ferri subcarbonas, ʒ iiss.

Ferri phosphas, ʒ iss.

Magnesia carb., ʒ ii.

Sodii chlorid., grs. lxx.

Cretæ prep., ʒ iii.

Aquæ, f. ʒ ii.

Mix.

Of which a tea-spoonful was taken thrice daily.

Under this treatment the patient regained his former stamina, and was discharged on May 9th.—*Medical and Surgical Reporter.*

